# P-1292. Impact of Discontinuation of Serum Antibody Test Reagents on the Diagnosis of Amebiasis in Japan (2013-2022)

**DOI:** 10.1093/ofid/ofae631.1473

**Published:** 2025-01-29

**Authors:** Yoshiro Hadano, Hirotake Mori, Satoshi Ohno

**Affiliations:** Shimane University Hospital, Izumo, Shimane, Japan; Juntendo University Faculty of Medicine, Bunkyo-ku, Tokyo, Japan; Shimane University Hospital, Izumo, Shimane, Japan

## Abstract

**Background:**

Amebiasis, a notifiable infectious disease in Japan, had shown an increasing trend in reported cases until 2017 when the insurance-covered serum antibody testing reagent was discontinued. This change raised concerns about the subsequent decline in reported cases. Our study aimed to investigate the impact of discontinuing serum antibody test reagent production on amebiasis diagnosis trends.

**Methods:**

We retrospectively analyzed amebiasis cases from January 2013 to December 2022 using the National Center for Epidemiology of Infectious Diseases system. We employed Poisson analysis to assess weekly amebiasis case trends before and after the discontinuation period, which we divided into pre discontinuation (2013-2017), discontinuation (2018-2019), and COVID-19 pandemic (2020-2022) phases.

**Results:**

A total of 8,907 cases of amebiasis were reported during the study period. The average weekly number of cases was 20.4 in 2014-2017, 16.4 in 2018-2019, and 10.7 in 2020-2022. After adjusting for the effects of the COVID-19 pandemic, the frequency of diagnosis decreased during the discontinuation period (prevalence rate ratio = 0.76; 95% CI, 0.66-0.86; P < 0.01), especially for extraintestinal amebiasis (prevalence rate ratio = 0.35; 95% CI, 0.22-0.57; P < 0.01) compared with intraintestinal amebiasis (prevalence rate ratio = 0.81; 95% CI, 0.71-0.93; P < 0.01).

**Conclusion:**

After adjusting for the effects of the COVID-19 pandemic, the number of reported amebiasis cases per week significantly decreased after discontinuation of the serum antibody test reagent, even during the COVID-19 pandemic phase in Japan. To ensure accurate diagnosis, the availability of antibody reagents for serum testing, covered by insurance, should be encouraged.

**Disclosures:**

**Satoshi Ohno, M.D. Ph.D.**, Otsuka Pharmaceutical Factory: Advisor/Consultant

